# Supplementary material for: Analyzing cancer gene expression data through the lens of normal tissue-specificity
Source: PLoS Comput Biol. 2021 Jun 18;17(6):e1009085. doi: 10.1371/journal.pcbi.1009085 (PMC8244857; doi:10.1371/journal.pcbi.1009085)
Supplement: S1 Text — The supporting information file includes supplemental methods and results (Tables A and B and Figures A-I, which are described below). Table A. TCGA cancer type statistics. The 21 analyzed TCGA cancer types, number of tumor samples with expression data, mean age, gender proportions, and corresponding HPA normal tissues. Note that gender proportions for some cancers may not add up to 1.0 if gender is unavailable for some samples. Table B. Distances between each cancer type and the corresponding normal tissue in reduced principal component (PC) space. The Euclidean distance was computed between the projections of each cancer type and the associated normal tissue in the space spanned by the all PC with non-zero variance of the mean gene expression matrix. The ‘Cancer/normal relative distance’ column contains the ratio of the distance between each cancer and normal tissue pair to the average distance between the cancer and all other cancers or normal tissues. The ‘Cancer/normal-specific relative distance’ column contains a similar distance ratio computed in the space spanned by the first two PCs of cancer-specific and normal tissue-specific mean expression values. The ‘Distance ratio’ column contains the ratio of the ‘Cancer/normal-specific relative distance’ to the ‘Cancer/normal relative distance’. Fig A. Variance of the PCs of the mean gene expression matrix. Fig B. Projection of TCGA cancer types and associated HPA normal tissues onto principal components 3–8 of the mean expression values. Principal components are computed from a matrix of mean gene expression values, i.e., a matrix containing all ci,j and ni.j statistics. a) Cancers are represented by blue points and are enclosed in the blue shaded region; normal tissues are represented by red points and are enclosed in the red shaded region. b) Each normal tissue and the associated cancer type(s) are enclosed in a separate shaded region. Fig C. Projection of TCGA cancer types and associated HPA normal tissues onto [file pcbi.1009085.s001.pdf]

# Supporting Information: Analyzing cancer gene expression data through the lens of normal tissue-specificity

H. Robert Frost

## Contents

**1 Supplemental Methods** **1**
1.1 Cancer and normal gene expression data . . . 1
1.2 Gene sets . . . 2
1.3 Generation of normal tissue ($n_{i,j}$) and cancer ($c_{i,j}$) mean expression . . . 2
1.4 Generation of normal tissue ($n^*_{i,j}$ and cancer ($c^*_{i,j}$) specificity . . . 2
1.5 Generation of cancer/normal relative expression ($r^*_{i,j}$) . . . 2
1.6 Generation of cancer survival association ($s^*_{i,j}$) . . . 3
1.7 Generation of Table 5 . . . 3
1.8 Generation of Figures 5, C and D . . . 3
1.9 Generation of Figures 6, E and F . . . 3

**2 Supplemental Results** **3**

## List of Tables

A **The 21 analyzed TCGA cancer types, number of tumor samples with expression data, mean age, gender proportions, and corresponding HPA normal tissues. Note that gender proportions for some cancers may not add up to 1.0 if gender is unavailable for some samples.** . . . 2
B **Distances between each cancer type and the corresponding normal tissue in reduced principal component (PC) space.** The Euclidean distance was computed between the projections of each cancer type and the associated normal tissue in the space spanned by the all PC with non-zero variance of the mean gene expression matrix. The 'Cancer/normal relative distance' column contains the ratio of the distance between each cancer and normal tissue pair to the average distance between the cancer and all other cancers or normal tissues. The 'Cancer/normal-specific relative distance' column contains a similar distance ratio computed in the space spanned by the first two PCs of cancer-specific and normal tissue-specific mean expression values. The 'Distance ratio' column contains the ratio of the 'Cancer/normal-specific relative distance' to the 'Cancer/normal relative distance'. . . . 7

## List of Figures

A **Variance of the PCs of the mean gene expression matrix.** . . . 4
B **Projection of TCGA cancer types and associated HPA normal tissues onto principal components 3-8 of the mean expression values.** Principal components are computed from a matrix of mean gene expression values, i.e., a matrix containing all $c_{i,j}$ and $n_{i.j}$ statistics. a) Cancers are represented by blue points and are enclosed in the blue shaded region; normal tissues are represented by red points and are enclosed in the red shaded region. b) Each normal tissue and the associated cancer type(s) are enclosed in a separate shaded region. . . . 5

C **Projection of TCGA cancer types and associated HPA normal tissues onto principal components of the tissue/cancer-specific mean expression values.** Principal components are computed from a matrix of mean gene expression values, i.e., a matrix containing all $c_{i,j}$ and $n_{i.j}$ statistics. a) Cancers are represented by blue points and are enclosed in the blue shaded region; normal tissues are represented by red points and are enclosed in the red shaded region. b) Each normal tissue and the associated cancer type(s) are enclosed in a separate shaded region. . . . . . . . 6
D **Association between normal tissue-specificity ($n^*_{i,j}$ statistics) on the y-axis and cancer/normal relative expression ($r_{i,j}$ statistics) on the x-axis.** Each point in both plots represents a single gene and the red lines reflect the linear regression fit. . . . . . . . . . . . . . . . . . . . . . 8
E **Association between normal tissue-specificity ($n^*_{i,j}$ statistics) on the y-axis and cancer survival ($s_{i,j}$ statistics) on the x-axis.** Each point in both plots represents a single gene and the red lines reflect the linear regression fit. . . . . . . . . . . . . . . . . . . . . . . . . . . . . . . . . . 9
F **Quantile-quantile plots illustrating the impact of filtering tissue-specific genes on gene set testing of MSigDB Hallmark pathways using cancer/normal relative expression ($r_{i,j}$).** Each plot contrasts the distribution of p-values (the black line) from tests for enrichment of large $r_{i,j}$ statistics among the gene set members, i.e., are gene set members more likely to be up-regulated in cancer vs. the normal tissue? The x-axis reflects the p-value distribution when all genes are included and the y-axis reflects the p-value distribution when genes are filtered according to normal tissue-specificity ($n^*_{i,j}$). Gene filtering removed the 20% of genes with the largest tissue-specificity values (i.e., keeps genes that are less tissue-specific). . . . . . . . . . . . . . . . . . . . . . . . . . . . . . 10
G **Quantile-quantile plots illustrating the impact of filtering non-tissue-specific genes on gene set testing of MSigDB Hallmark pathways using cancer/normal relative expression ($r_{i,j}$).** Each plot contrasts the distribution of p-values (the black line) from tests for enrichment of small $r_{i,j}$ statistics among the gene set members, i.e., are gene set members more likely to be down-regulated in cancer vs. the normal tissue? The x-axis reflects the p-value distribution when all genes are included and the y-axis reflects the p-value distribution when genes are filtered according to normal tissue-specificity ($n^*_{i,j}$). Gene filtering removed the 20% of genes with the smallest tissue-specificity values (i.e., keeps genes that are more tissue-specific). . . . . . . . . . . . . . . . . . . . . . . . . . . 11
H **Quantile-quantile plots illustrating the favorable impact of tissue-specific gene filtering on cancer survival analysis.** Each plot contrasts the distribution of p-values (the black line) capturing favorable prognostic status of all genes for genes filtered according to tissue-specificity. The x-axis reflects the p-value distribution when all genes are included and the y-axis reflects the p-value distribution when genes are filtered according to normal tissue-specificity ($n^*_{i,j}$). Gene filtering removed genes where $n^*_{i,j} < log(0.8)$ (i.e., removed genes expressed at a lower level in the associated normal tissue than in the average tissue). . . . . . . . . . . . . . . . . . . . . . . . . . . . . . . . . . . . . . 12
I **Quantile-quantile plots illustrating unfavorable impact of tissue-specific gene filtering on cancer survival analysis.** Each plot contrasts the distribution of p-values (the black line) capturing unfavorable prognostic status of all genes for genes filtered according to tissue-specificity. The x-axis reflects the p-value distribution when all genes are included and the y-axis reflects the p-value distribution when genes are filtered according to normal tissue-specificity ($n^*_{i,j}$). Gene filtering removed genes where $n^*_{i,j} > log(1.2)$ (i.e., removed genes expressed at a higher level in the associated normal tissue than in the average tissue). . . . . . . . . . . . . . . . . . . . . . . . . . . . . . . . . . . . . . 13

# 1 Supplemental Methods

A companion website for this paper can be found at https://hrfrost.host.dartmouth.edu/CancerNormal/. This site provides access to files containing all of the gene-level statistics used to compute the results (i.e., the $n_{i,j}, c_{i,j}, n^*_{i,j}$, and $c^*_{i,j}$ statistics) and the version of the Human Protein Atlas (HPA) normal tissue gene expression data ("HPA.normal.FPKM.GDCpipeline.csv") that was specially normalized by the HPA group as FPKM using a pipeline similar to that employed by GDC for the TCGA data (this data was generated for the "Human Pathology Atlas" paper [1]).

## 1.1 Cancer and normal gene expression data

The activity of human protein-coding genes in 21 common cancers and 18 associated normal tissues was determined using transcriptomic data from The Cancer Genome Atlas (TCGA) [2] and transcriptomic data from the Human Protein Atlas (HPA) [3]. Table A below lists the 21 supported TCGA cancer types, number of tumor samples,

matching HPA cancer types and associated HPA normal human tissue types. As detailed in the HPA paper [3], the HPA RNA-seq measurements were made on frozen tissue sections from the Uppsala Biobank for three healthy individuals. These 21 cancer types were selected based on the availability of gene expression data for the corresponding normal tissues in the HPA. A similar set of 17 TCGA cancer types were selected for the analysis by Uhlen et al. [1]. In contrast to Uhlen et al., we have separately analyzed the three renal cancer types (kidney chromophobe, kidney renal clear cell carcinoma, and kidney renal papillary cell carcinoma), separately analyzed colon cancer and rectal cancer, and separately analyzed lung adenocarcinoma and lung squamous cell carcinoma. RNA-seq data was filtered to include just the 19,670 genes with measurements on all normal tissues and cancer types and was normalized as FPKM +1 using the Genome Data Commons (GDC) pipeline for the TCGA data and using a pipeline similar to GDC pipeline for the HPA data.

- For TCGA, the PANCAN RNA-seq and phenotype data contained in the files "GDC-PANCAN.htseq_fpkm.tsv.gz" and "GDC-PANCAN.basic_phenotype.tsv" was accessed from the GCD Data Portal and can be downloaded from https://gdc-hub.s3.us-east-1.amazonaws.com/download/GDC-PANCAN.htseq_fpk and https://gdc-hub.s3.us-east-1.amazonaws.com/download/GDC-PANCAN.basic_phenotype.tsv.gz.
- For HPA, the HPA staff provided normal tissue gene expression data in the file "HPA.normal.FPKM.GDCpipeline.csv"; this data was specially normalized by the HPA group as FPKM using a pipeline similar to that employed by GDC for the TCGA data (this data was generated for the "Human Pathology Atlas" paper [1]). A copy of this file can be retrieved from the paper website (https://hrfrost.host.dartmouth.edu/CancerNormal/).
- Prognostic p-vlaues for each gene in each cancer type were retrieved from the HPA file "pathology.tsv.zip" downloaded from https://www.proteinatlas.org/download/pathology.tsv.zip.

| TCGA abbrev. | # TCGA samples | Mean TCGA age at diagnosis (sd) | TCGA male/female proportions | HPA cancer type | HPA tissue |
|---|---|---|---|---|---|
| BLCA | 430 | 68.2 (10.5) | 0.73/0.27 | Bladder Urothelial Carcinoma | urinary bladder |
| BRCA | 1,217 | 58.3 (13.4) | 0.01/0.99 | Breast Invasive Carcinoma | breast |
| CESC | 309 | 48.2 (13.7) | 0/1 | Cervical Squamous Cell Carcinoma and ... | cervix, uterine |
| COAD | 512 | 67.6 (13.0) | 0.52/0.48 | Colon Adenocarcinoma | colon |
| GBM | 173 | 57.7 (14.3) | 0.60/0.37 | Glioblastoma Multiforme | cerebral cortex |
| HNSC | 546 | 61.0 (11.9) | 0.73/0.27 | Head and Neck Squamous Cell Carcinoma | |
| KICH | 89 | 51.9 (14.3) | 0.55/0.45 | Kidney Chromophobe | kidney |
| KIRC | 607 | 60.8 (12.1) | 0.65/0.35 | Kidney Renal Clear Cell Carcinoma | kidney |
| KIRP | 321 | 62.0 (12.2) | 0.74/0.26 | Kidney Renal Papillary Cell Carcinoma | kidney |
| LIHC | 424 | 60.3 (13.8) | 0.66/0.34 | Liver Hepatocellular Carcinoma | liver |
| LUAD | 585 | 65.2 (10.1) | 0.39/0.47 | Lung Adenocarcinoma | lung |
| LUSC | 550 | 67.3 (8.7) | 0.73/0.27 | Lung Squamous Cell Carcinoma | lung |
| OV | 379 | 59.6 (11.4) | 0/0.97 | Ovarian Serous Cystadenocarcinoma | ovary |
| PAAD | 182 | 64.9 (11.4) | 0.56/0.44 | Pancreatic Adenocarcinoma | pancreas |
| PRAD | 551 | 60.9 (6.8) | 1/0 | Prostate Adenocarcinoma | prostate |
| READ | 177 | 64.2 (12.2) | 0.52/0.46 | Rectum Adenocarcinoma | rectum |
| SKCM | 472 | 58.2 (15.6) | 0.62/0.38 | Skin Cutaneous Melanoma | skin |
| STAD | 407 | 66.0 (10.7) | 0.63/0.37 | Stomach Adenocarcinoma | stomach |
| TGCT | 156 | 31.9 (9.2) | 0.89/0 | Testicular Germ Cell Tumors | testis |
| THCA | 568 | 47.2 (15.8) | 0.27/0.73 | Thyroid Carcinoma | thyroid gland |
| UCEC | 583 | 70.1 (9.6) | 0/1 | Uterine Corpus Endometrial Carcinoma | endometrium |

Table A: **The 21 analyzed TCGA cancer types, number of tumor samples with expression data, mean age, gender proportions, and corresponding HPA normal tissues. Note that gender proportions for some cancers may not add up to 1.0 if gender is unavailable for some samples.**

## 1.2 Gene sets

The Hallmark collection pathways were downloaded from version 7.0 of the Molecular Signatures Database (MSigDB) [4] (as downloaded from http://software.broadinstitute.org/gsea/downloads.jsp). Gene set testing of the Hallmark

pathways was performed using the pre-ranked version of the CAMERA method [5] (the *cameraPR()* R function in the *limma* package [6]).

### 1.3 Generation of normal tissue ($n_{i,j}$) and cancer ($c_{i,j}$) mean expression

The $c_{i,j}$ and $n_{i,j}$ statistics are simply computed as the mean of the FPKM values for gene $i$ across all samples profiled for TCGA cancer type $j$ for $c_{i,j}$ or the normal tissue associated with cancer type $j$ for $n_{i,j}$.

### 1.4 Generation of normal tissue ($n^*_{i,j}$ and cancer ($c^*_{i,j}$) specificity

The $c^*_{i,j}$ and $n^*_{i,j}$ statistics are computed as the log2 fold-change in mean expression for gene $i$ in cancer type $j$ or the associated normal tissue relative to the average for all cancer types or normal tissues. Specifically:

$$c^*_{i,j} = \log_2 \left(\frac{c_{i,j}}{1/21 \sum_{k=1}^{21} c_{i,k}}\right)$$
$$n^*_{i,j} = \log_2 \left(\frac{n_{i,j}}{1/18 \sum_{k=1}^{18} n_{i,k}}\right)$$

### 1.5 Generation of cancer/normal relative expression ($r^*_{i,j}$)

The $r_{i,j}$ is computed as the log2 fold-change between mean expression in cancer type $j$ and mean expression in the associated normal tissue, i.e., $r_{i,j} = \log_2(c_{i,j}/n_{i,j})$.

### 1.6 Generation of cancer survival association ($s^*_{i,j}$)

The $s_{i,j}$ statistics represents a signed log p-value where the p-value is generated via a Kaplan-Meir (KM) test of the association between expression of gene $i$ and survival for cancer type $j$ as computed by Uhlen et al. [1]). As detailed in Uhlen et al., the gene expression values where discretized according to multiple thresholds and the threshold generating the most significant KM test was used to compute the p-value. If the association between gene expression and survival is favorable (i.e., higher expression of the gene is associated with better survival), the -log of the p-value is used to produce a positive statistic. If the association between gene expression and survival is unfavorable (i.e., higher expression of the gene is associated with worse survival), the log of the p-value is used to produce a negative statistic.

### 1.7 Generation of Table 5

To generate Table 5, gene set testing was performed using the pre-ranked version of the CAMERA method [5] (the *cameraPR()* R function in the *limma* package [6]) and the Hallmark pathway collection from MSigDB [4]. The relative expression between liver and cerebral cortex (i.e., $n_{i,\text{liver}}/n_{i,\text{cortex}}$) or between liver cancer and glioblastoma (i.e., $c_{i,\text{LIHC}}/c_{i,\text{GBM}}$) were used as the gene-level statistics for execution of *cameraPR()* with *use.ranks=T* and *inter.gene.cor=0*. For the first two columns, all genes present in both tissue types or cancer types were used. For the third column, filtering was performed to remove all genes whose fold-change in expression between liver and cerebral cortex was $\leq 0.5$ or $\geq 2$.

### 1.8 Generation of Figures 5, C and D

To generate the quantile-quantile (Q-Q) plots in Figures 5, C and D, gene set testing was performed using the pre-ranked version of the CAMERA method [5] (the *cameraPR()* R function in the *limma* package [6]) and the Hallmark pathway collection from MSigDB [4]. The log2 fold-change in expression between each cancer and the associated normal tissue (i.e, $r_{i,j}$ statistics) were used as the gene-level statistics for execution of *cameraPR()* with *use.ranks=F* and *inter.gene.cor=0.01*. Figure 5 contrasts the distribution of p-values (the black line) from tests for all 50 Hallmark pathways for each of the 21 cancer/normal pairs (total of 1050 tests) using all genes or genes filtered according to tissue-specificity. Figures C and D are generated using just the 50 p-values for a single cancer type. Panel a) in Figure 5 and the panels in Figure C contain the results from tests for enrichment of large $r_{i,j}$ statistics among the gene set members (i.e., are gene set members more likely to be up-regulated in cancer vs. the normal tissue?) and gene filtering removed the 20% of genes with the largest tissue-specificity values (i.e., kept genes that are less tissue-specific). Panel b) in Figure 5 and the panels in Figure S4 contain the results from tests for enrichment

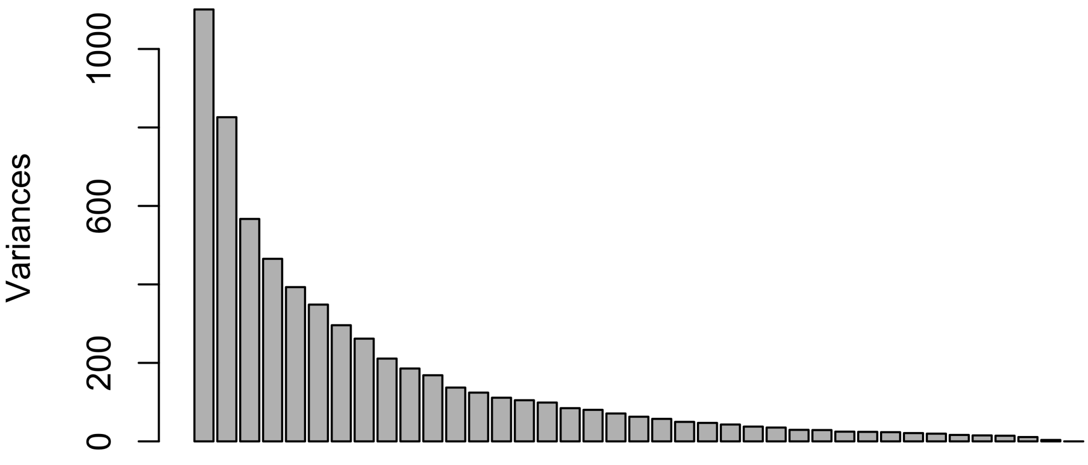


Figure A: **Variance of the PCs of the mean gene expression matrix.**

of small $r_{i,j}$ statistics among the gene set members (i.e., are gene set members more likely to be down-regulated in the cancer vs. the normal tissue?) and gene filtering removed the 20% of genes with the smallest tissue-specificity values (i.e., kept genes that are more tissue-specific). For all Q-Q plots, the p-values for gene sets whose direction of enrichment is opposite the target direction were set to 1.0, which causes the vertical portion of the black line.

### 1.9 Generation of Figures 6, E and F

The Q-Q plots in Figures 6, E and F contrast the distribution of filtered vs unfiltered p-values generated via Kaplan-Meir (KM) tests of the association between gene expression and cancer survival following the approach of Ulhen et al. [1]. For the analysis of favorably prognostic genes (panel a in Figure 6 and all panels in Figure E), the p-values for all unfavorable genes were set to 1.0 and filtering was performed to remove genes where $n^*_{i,j} < log(0.8)$, i.e., genes down-regulated in the associated tissue. For the analysis of unfavorably prognostic genes (panel b in Figure 6 and all panels in Figure F), the p-values for all favorable genes were set to 1.0 and filtering was performed to remove genes where $n^*_{i,j} > log(1.2)$, i.e., genes up-regulated in the associated tissue.

## 2 Supplemental Results

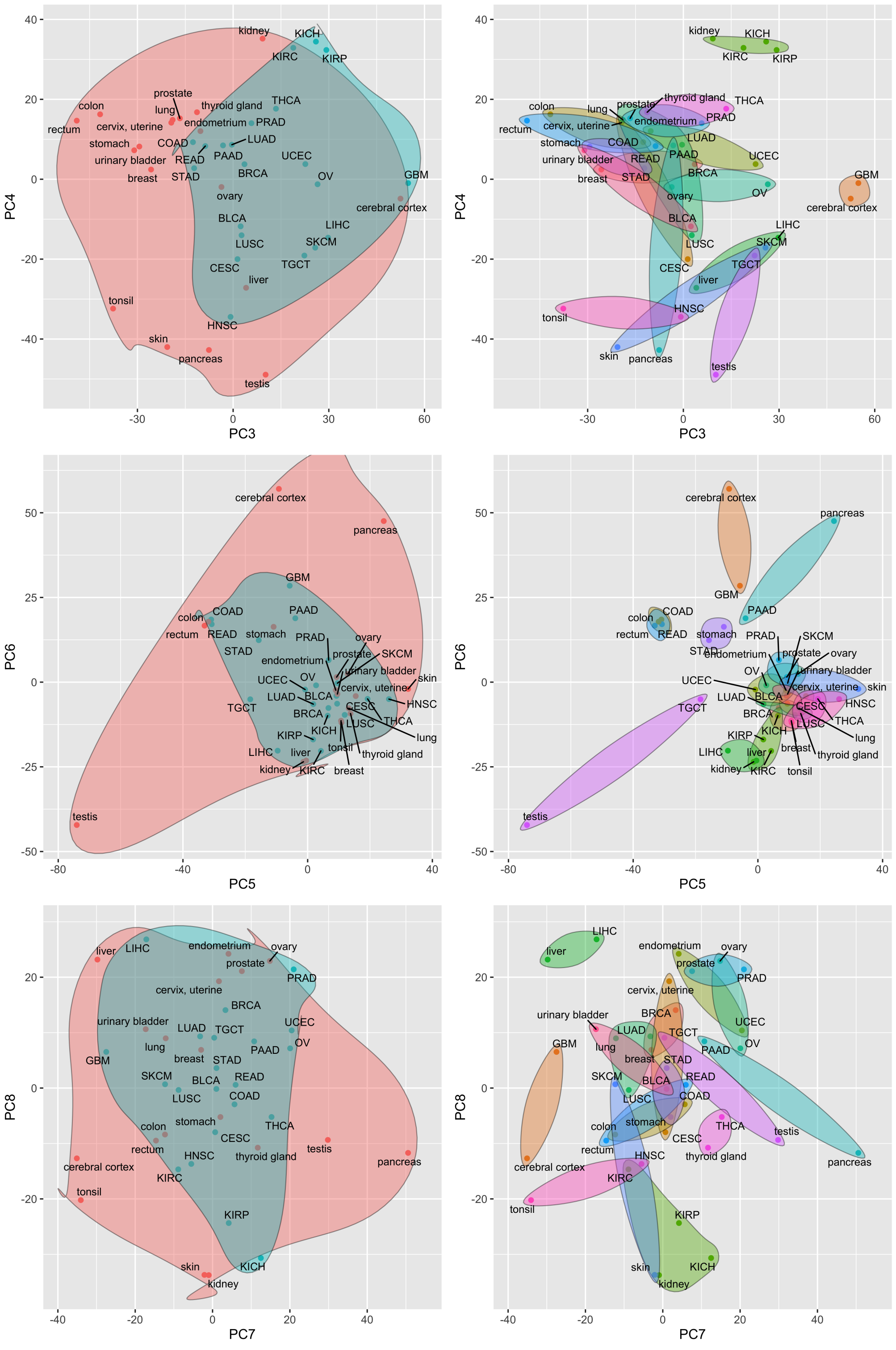


Figure B: **Projection of TCGA cancer types and associated HPA normal tissues onto principal components 3-8 of the mean expression values.** Principal components are computed from a matrix of mean gene expression values, i.e., a matrix containing all $c_{i,j}$ and $n_{i.j}$ statistics. a) Cancers are represented by blue points and are enclosed in the blue shaded region; normal tissues are represented by red points and are enclosed in the red shaded region. b) Each normal tissue and the associated cancer type(s) are enclosed in a separate shaded region.

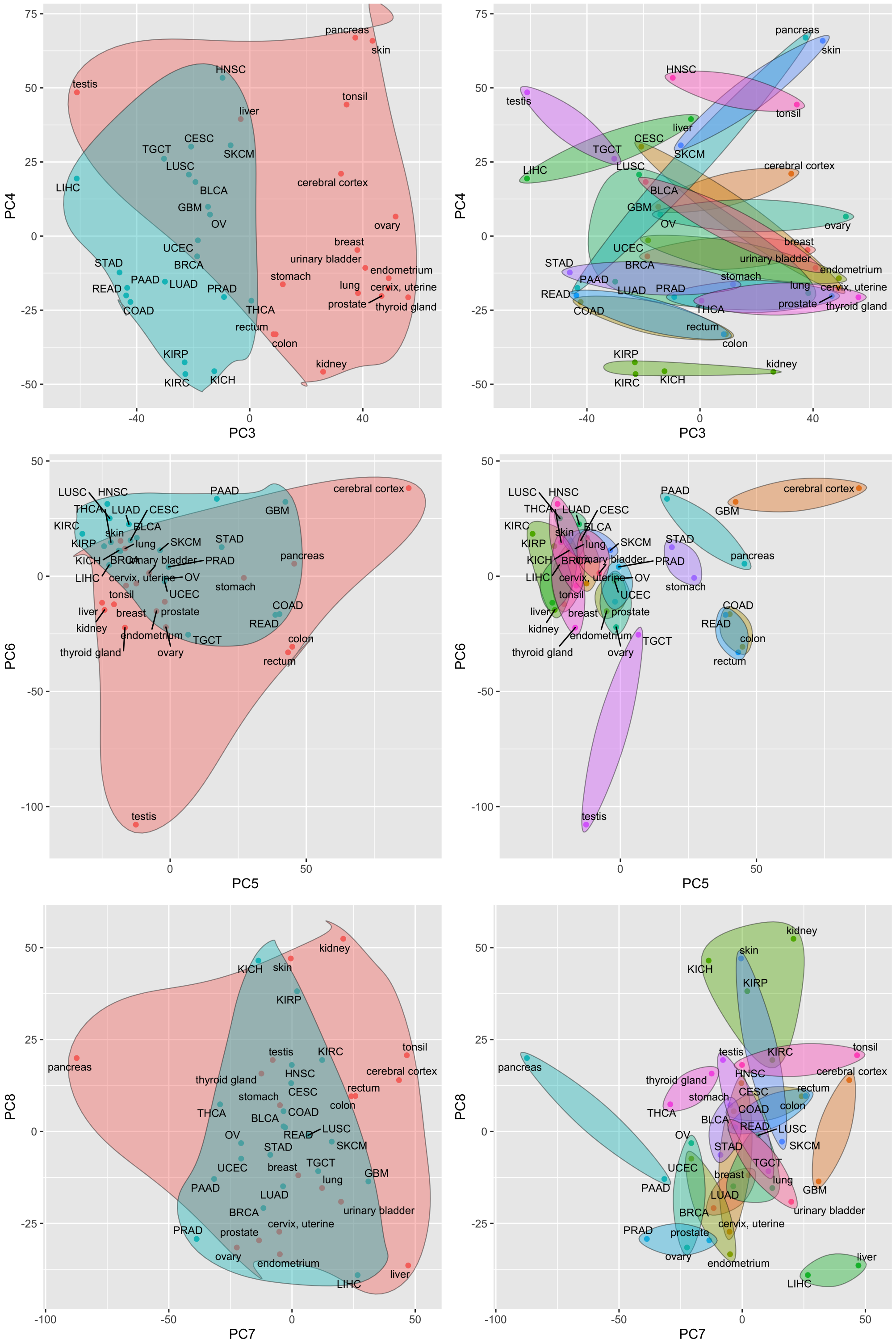

Figure C: **Projection of TCGA cancer types and associated HPA normal tissues onto principal components of the tissue/cancer-specific mean expression values.** Principal components are computed from a matrix of mean gene expression values, i.e., a matrix containing all $c_{i,j}$ and $n_{i.j}$ statistics. a) Cancers are represented by blue points and are enclosed in the blue shaded region; normal tissues are represented by red points and are enclosed in the red shaded region. b) Each normal tissue and the associated cancer type(s) are enclosed in a separate shaded region.

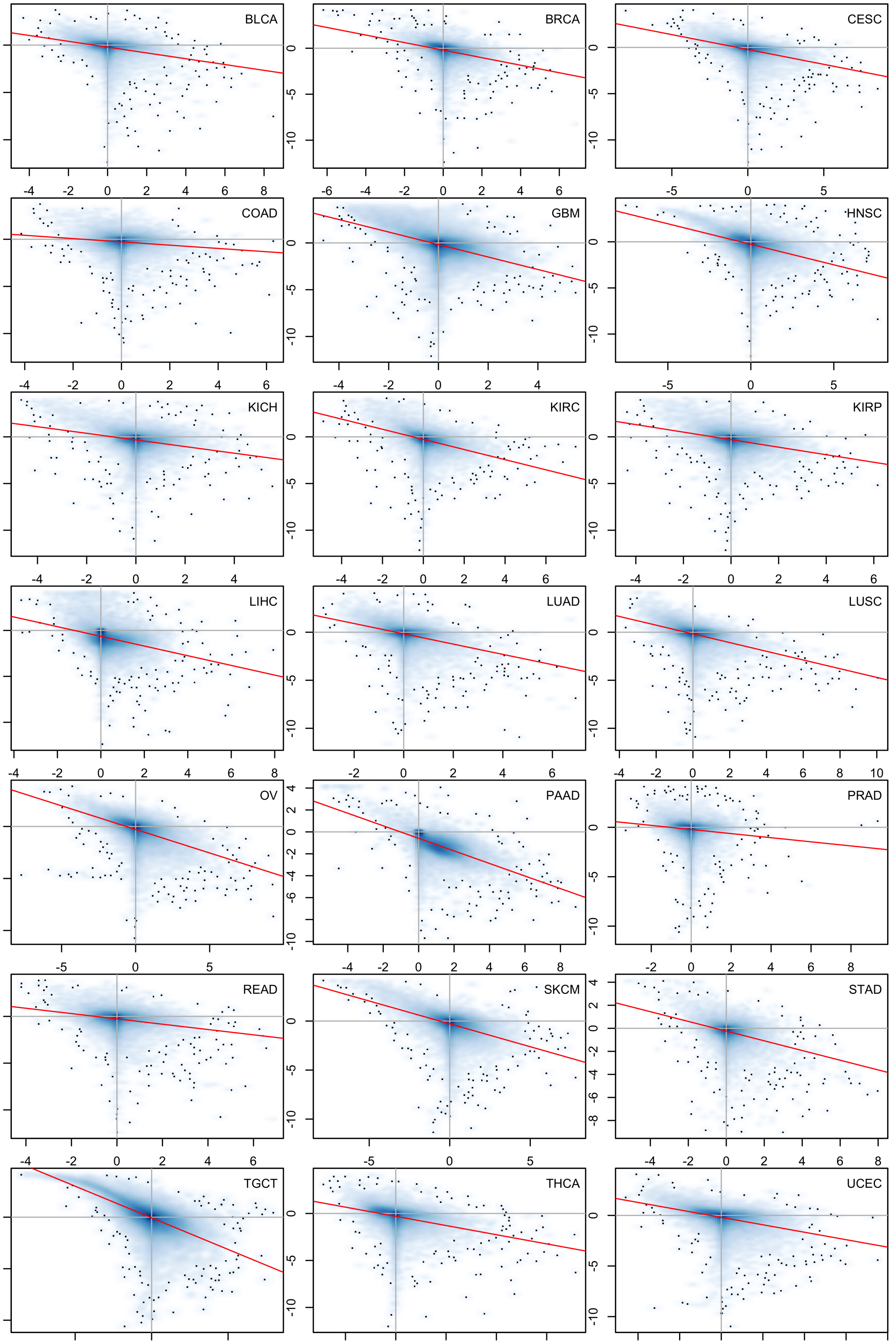


Figure D: **Association between normal tissue-specificity ($n^*_{i,j}$ statistics) on the y-axis and cancer/normal relative expression ($r_{i,j}$ statistics) on the x-axis.** Each point in both plots represents a single gene and the red lines reflect the linear regression fit.

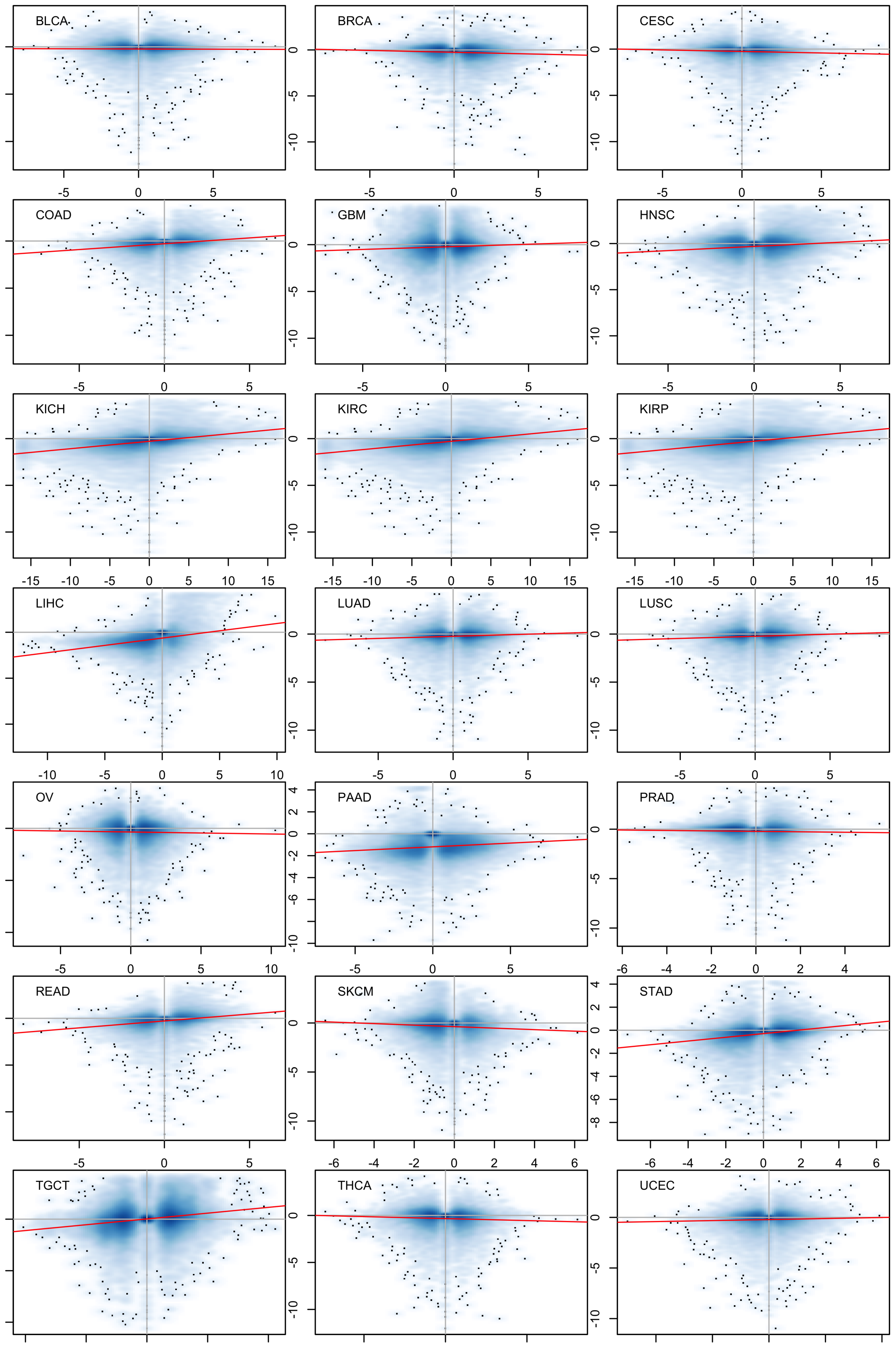


Figure E: **Association between normal tissue-specificity ($n^*_{i,j}$ statistics) on the y-axis and cancer survival ($s_{i,j}$ statistics) on the x-axis.** Each point in both plots represents a single gene and the red lines reflect the linear regression fit.

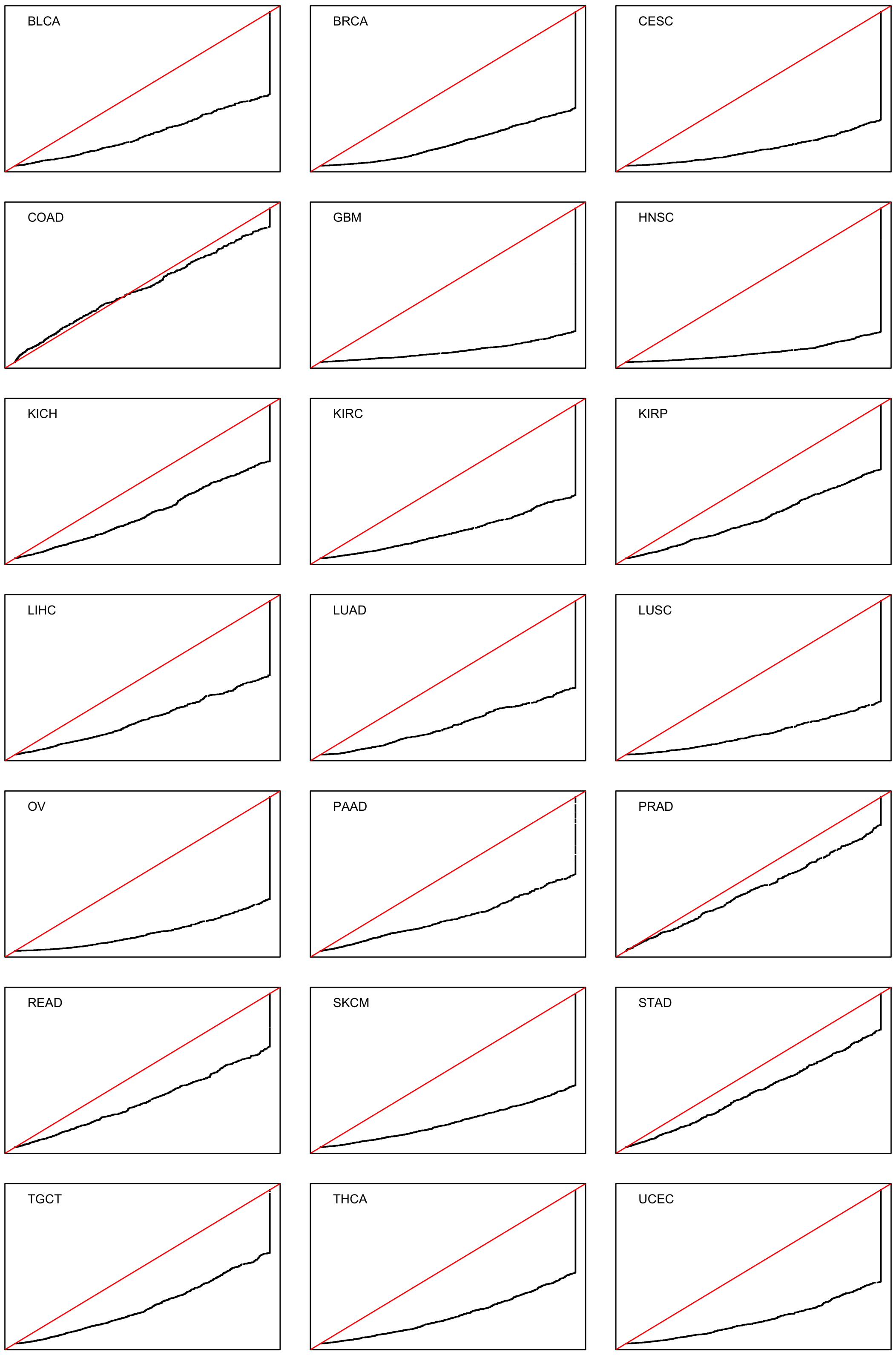


Figure F: **Quantile-quantile plots illustrating the impact of filtering tissue-specific genes on gene set testing of MSigDB Hallmark pathways using cancer/normal relative expression ($r_{i,j}$).** Each plot contrasts the distribution of p-values (the black line) from tests for enrichment of large $r_{i,j}$ statistics among the gene set members, i.e., are gene set members more likely to be up-regulated in cancer vs. the normal tissue? The x-axis reflects the p-value distribution when all genes are included and the y-axis reflects the p-value distribution when genes are filtered according to normal tissue-specificity ($n^*_{i,j}$). Gene filtering removed the 20% of genes with the largest tissue-specificity values (i.e., keeps genes that are less tissue-specific).

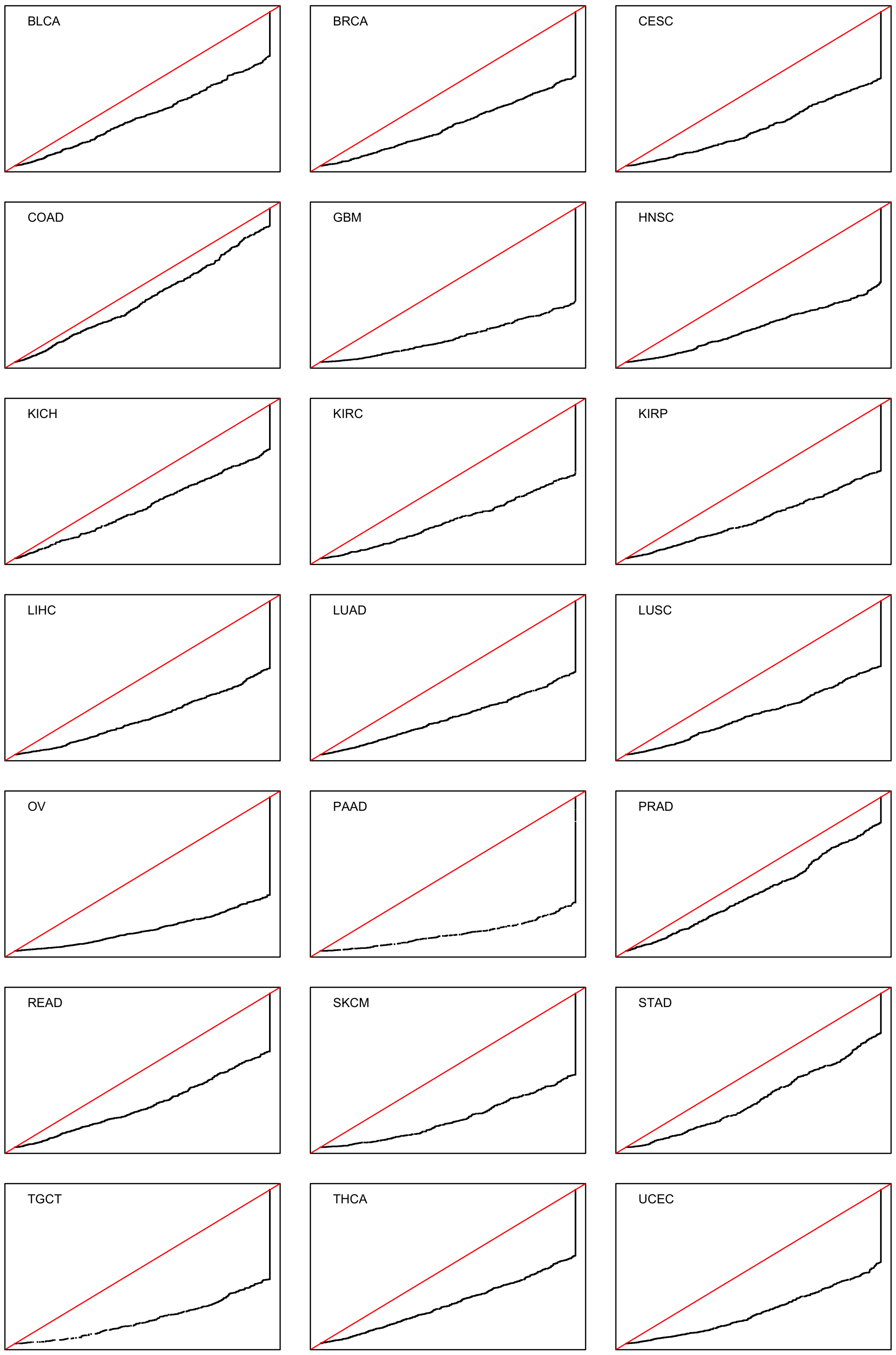


Figure G: **Quantile-quantile plots illustrating the impact of filtering non-tissue-specific genes on gene set testing of MSigDB Hallmark pathways using cancer/normal relative expression ($r_{i,j}$).** Each plot contrasts the distribution of p-values (the black line) from tests for enrichment of small $r_{i,j}$ statistics among the gene set members, i.e., are gene set members more likely to be down-regulated in cancer vs. the normal tissue? The x-axis reflects the p-value distribution when all genes are included and the y-axis reflects the p-value distribution when genes are filtered according to normal tissue-specificity ($n^*_{i,j}$). Gene filtering removed the 20% of genes with the smallest tissue-specificity values (i.e., keeps genes that are more tissue-specific).

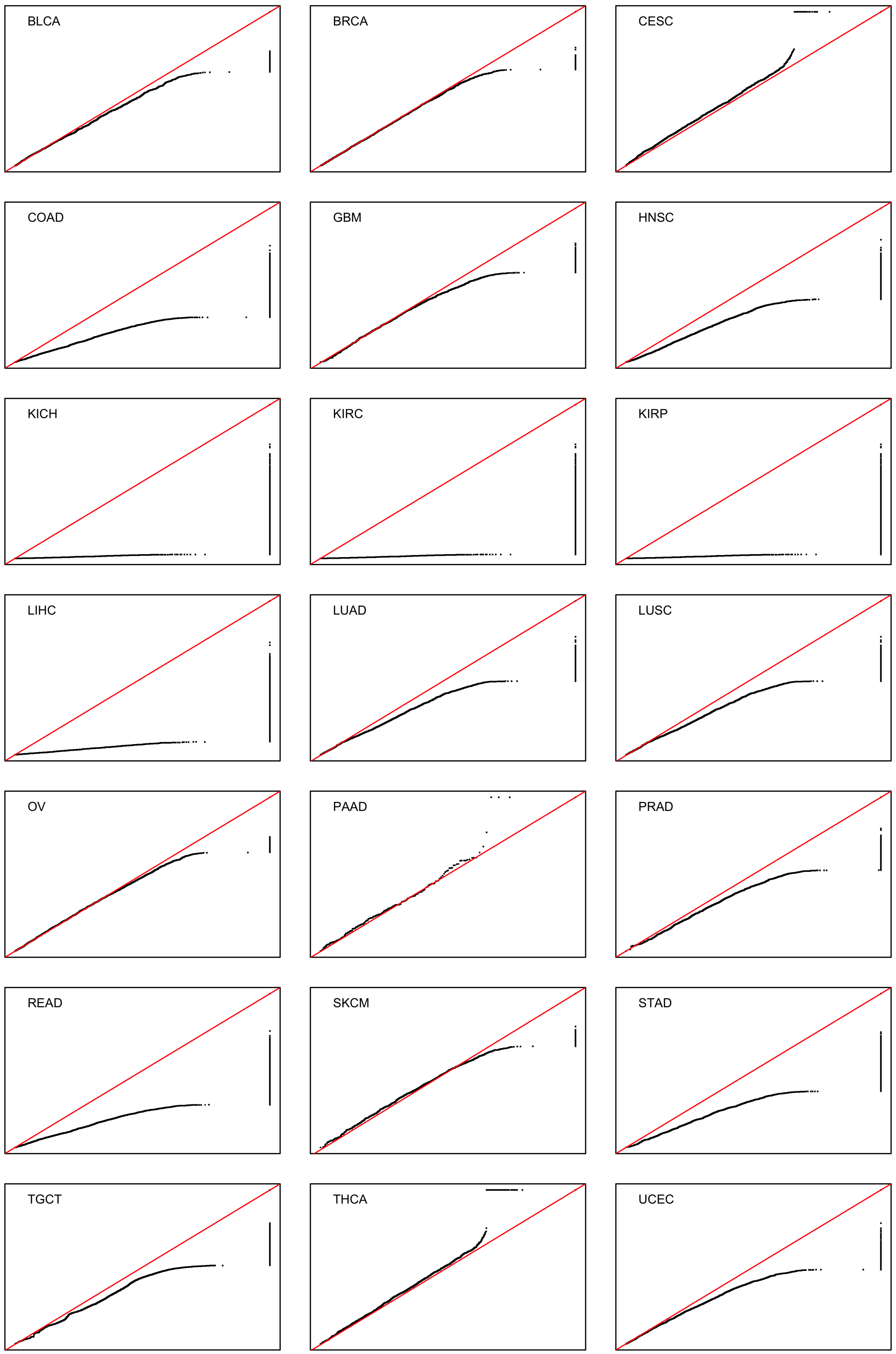


Figure H: **Quantile-quantile plots illustrating the favorable impact of tissue-specific gene filtering on cancer survival analysis.** Each plot contrasts the distribution of p-values (the black line) capturing favorable prognostic status of all genes for genes filtered according to tissue-specificity. The x-axis reflects the p-value distribution when all genes are included and the y-axis reflects the p-value distribution when genes are filtered according to normal tissue-specificity ($n^*_{i,j}$). Gene filtering removed genes where $n^*_{i,j} < log(0.8)$ (i.e., removed genes expressed at a lower level in the associated normal tissue than in the average tissue).

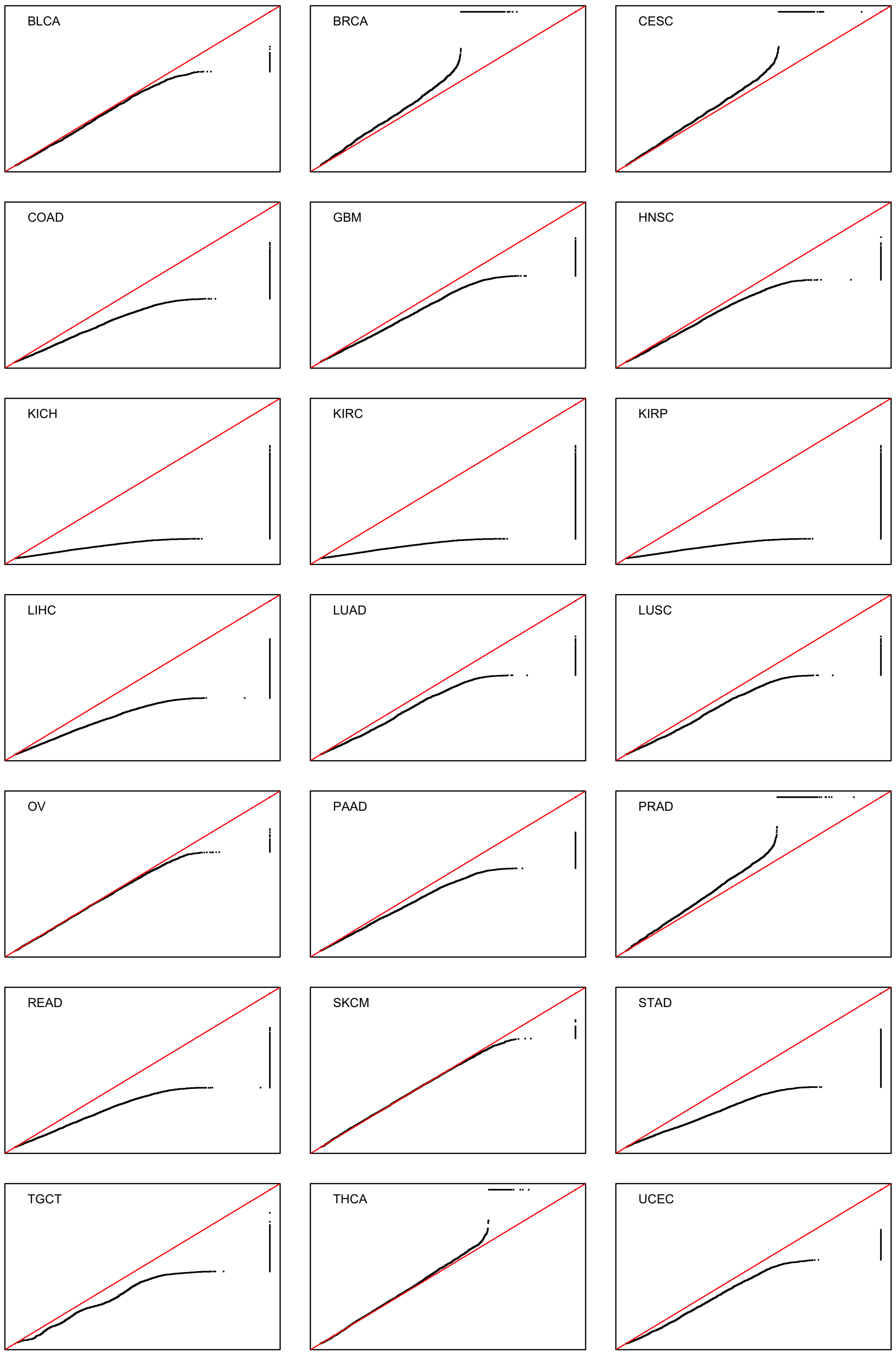


Figure I: **Quantile-quantile plots illustrating unfavorable impact of tissue-specific gene filtering on cancer survival analysis.** Each plot contrasts the distribution of p-values (the black line) capturing unfavorable prognostic status of all genes for genes filtered according to tissue-specificity. The x-axis reflects the p-value distribution when all genes are included and the y-axis reflects the p-value distribution when genes are filtered according to normal tissue-specificity ($n^*_{i,j}$). Gene filtering removed genes where $n^*_{i,j} > log(1.2)$ (i.e., removed genes expressed at a higher level in the associated normal tissue than in the average tissue).

| TCGA abbrev. | HPA tissue | Cancer/normal relative distance | Cancer/normal-specific relative distance | Distance ratio |
|---|---|---|---|---|
| BLCA | urinary bladder | 0.856 | 0.776 | 0.906 |
| BRCA | breast | 0.773 | 0.717 | 0.927 |
| CESC | cervix, uterine | 1.03 | 0.934 | 0.906 |
| COAD | colon | 0.706 | 0.636 | 0.9 |
| GBM | cerebral cortex | 0.761 | 0.735 | 0.966 |
| HNSC | tonsil | 0.943 | 0.898 | 0.952 |
| KICH | kidney | 0.689 | 0.729 | 1.06 |
| KIRC | kidney | 0.733 | 0.801 | 1.09 |
| KIRP | kidney | 0.746 | 0.731 | 0.98 |
| LIHC | liver | 0.606 | 0.566 | 0.934 |
| LUAD | lung | 0.75 | 0.764 | 1.02 |
| LUSC | lung | 0.927 | 0.904 | 0.976 |
| OV | ovary | 1.14 | 1.01 | 0.881 |
| PAAD | pancreas | 1.42 | 1.4 | 0.984 |
| PRAD | prostate | 0.598 | 0.608 | 1.02 |
| READ | rectum | 0.791 | 0.683 | 0.863 |
| SKCM | skin | 1.04 | 1.07 | 1.02 |
| STAD | stomach | 0.762 | 0.791 | 1.04 |
| TGCT | testis | 1.27 | 1.07 | 0.84 |
| THCA | thyroid gland | 0.704 | 0.681 | 0.967 |
| UCEC | endometrium | 0.988 | 0.854 | 0.864 |

Table B: **Distances between each cancer type and the corresponding normal tissue in reduced principal component (PC) space.** The Euclidean distance was computed between the projections of each cancer type and the associated normal tissue in the space spanned by the all PC with non-zero variance of the mean gene expression matrix. The 'Cancer/normal relative distance' column contains the ratio of the distance between each cancer and normal tissue pair to the average distance between the cancer and all other cancers or normal tissues. The 'Cancer/normal-specific relative distance' column contains a similar distance ratio computed in the space spanned by the first two PCs of cancer-specific and normal tissue-specific mean expression values. The 'Distance ratio' column contains the ratio of the 'Cancer/normal-specific relative distance' to the 'Cancer/normal relative distance'.

# References


[1] Uhlen M, Zhang C, Lee S, Sjöstedt E, Fagerberg L, Bidkhori G, et al. A pathology atlas of the human cancer transcriptome. Science. 2017;357(6352). doi:10.1126/science.aan2507.

[2] Cancer Genome Atlas Research Network, Weinstein JN, Collisson EA, Mills GB, Shaw KRM, Ozenberger BA, et al. The Cancer Genome Atlas Pan-Cancer analysis project. Nat Genet. 2013;45(10):1113–20. doi:10.1038/ng.2764.

[3] Uhlén M, Fagerberg L, Hallström BM, Lindskog C, Oksvold P, Mardinoglu A, et al. Proteomics. Tissue-based map of the human proteome. Science. 2015;347(6220):1260419. doi:10.1126/science.1260419.

[4] Liberzon A, Subramanian A, Pinchback R, Thorvaldsdóttir H, Tamayo P, Mesirov JP. Molecular signatures database (MSigDB) 3.0. Bioinformatics. 2011;27(12):1739–40. doi:10.1093/bioinformatics/btr260.

[5] Wu D, Smyth GK. Camera: a competitive gene set test accounting for inter-gene correlation. Nucleic Acids Research. 2012;40(17):e133. doi:10.1093/nar/gks461.

[6] Ritchie ME, Phipson B, Wu D, Hu Y, Law CW, Shi W, et al. limma powers differential expression analyses for RNA-sequencing and microarray studies. Nucleic Acids Res. 2015;43(7):e47. doi:10.1093/nar/gkv007.
