# Supplementary material for: Analyzing cancer gene expression data through the lens of normal tissue-specificity
Source: PLoS Comput Biol. 2021 Jun 18;17(6):e1009085. doi: 10.1371/journal.pcbi.1009085 (PMC8244857; doi:10.1371/journal.pcbi.1009085)
Supplement: Attachment — Submitted filename: Frost_CancerAndTissueSpecificity_Response_to_Comments.pdf [file pcbi.1009085.s002.pdf]

Response to reviewer comments for "Analyzing cancer gene expression data through the lens of normal tissue-specificity"

H. Robert Frost

We appreciate the thoughtful, detailed and very helpful feedback from the editors and reviewers. Responses to each of the editor's and reviewer's comments are outlined in the sections below (comments are in bold and our response follows in normal font). In addition to the revised manuscript and SI, the resubmission includes files showing the differences between these revised versions and the original versions, as computed via latexdiff. We have also created a paper-associated website (https://hrfrost.host.dartmouth.edu/CancerNormal/) that provides access to files containing all of the gene-level statistics used to compute the results (i.e., the $n_{i,j}, c_{i,j}, n^*_{i,j}$, and $c^*_{i,j}$ statistics) and the version of the Human Protein Atlas (HPA) normal tissue gene expression data ("HPA.normal.FPKM.GDCpipeline.csv") that was specially normalized by the HPA group as FPKM using a pipeline similar to that employed by GDC for the TCGA data (this data was generated for the "Human Pathology Atlas" paper [1]).

We believe that addressing the editor's reviewer's comments has substantially improved the revised manuscript by:

- Clarifying the computation and biological interpretation of the tissue-specific and cancer-specific gene expression statistics $n*_{ij}$ and $c*_{ij}$.
- Clarifying the motivation for the PCA-based analysis and providing visualization and distance results for additional PCs.
- Including inference results for the correlation-based statistics.
- Expanding the bibliography.
- Providing access to the gene-level statistics used to compute the results and the specially processed HPA RNA-seq data.

1 Editor Comments

I think this study has potential. In the literature, only a handful of studies have paid sufficient attention to gene expression data of normal tissues. The paper is overall well written (although reviewers have noticed a few typos and small mistakes). The analysis is mostly rigorous, and the findings can be potentially informative.

1. **Limited literature is cited. This can be improved to better position this study.**

   We agree with the editor that the currently bibliography is limited, which is due in part to the limited attention this specific topic has received in the cancer genomics field. To address this, we have included several additional references in the background related to the tissue-specificity of cancer.

2. **A possibly minor technical question: when constructing the PCs, are all genes used? Most genes are not cancer related, and including all (which contain a huge amount of noises) can be problematic.**

   We thank the editor for raising this question. When constructing the PCs, we used all genes, which is consistent with both the approach taken by Uhlen et al. [1] for a similar PC analysis and the goal of our PC analysis to highlight the genome-wide transcriptional differences/similarities between cancers and associated tissues.

3. **Why the first two PCs? Statistically and biologically, there is no reason why, for example, the second PC is more important than the third one.**

   The editor raises an excellent point which was also identified by reviewer 1 (see major comment #2). The projection onto the first 2 PCs was used for consistency with the similar analysis by Uhlen et al. [1]. While the first 2 PCs do explain more gene expression variance than any other pair of PCs, the editor makes a good point that other directions also have biological relevance. To address this comment, we have included in the supplemental material projections onto PCs 3-8 (for both the unadjusted mean expression matrix and the tissue/cancer-specific mean expression matrix), a scree plot showing all PC variances and a version of Table 3 where the Euclidean distances are computed on all PCs with non-zero variance. As seen from these additional results, cancers and associated normal tissues differ primarily along the directions of the 1st and 3rd PCs, which is consistent with the findings in Table 2 regarding the overall similarity between the transcriptomes of cancers and associated normal tissues. We have updated the relevant text in the main manuscript to clarify the interpretation of the projection onto the first 2 PCs and mention that results for additional PCs can be found in the supplement.

4. **I understand the importance of looking into gene expressions of normal tissues. However, it is still unclear to me how I can potentially use findings in this article in translational/clinical studies. This aspect needs to be significantly strengthened.**

   We appreciate the editor raising this concern and have include a new subsection under "Results and discussion" named "Guidance on how to leverage normal tissue-specific associations for cancer transcriptomic analyses" that provides more specific guidance on how researchers can apply these findings for their own data analysis projects. In brief, the findings in this paper can be leveraged by researchers to filter/weight genes according to normal tissue-specificity (i.e., the $n*_{i,j}$ statistics) for analyses based on cancer gene expression data. The scenarios supported by the findings in this paper include survival analysis, comparative analysis of different cancers, and comparative analysis of cancer and normal tissue. It is important to note that these scenarios correspond primarily to hypothesis generating/exploratory investigations rather than to translational/clinical studies. The translational impact of these findings is likely to be indirect, i.e., researchers leverage tissue-specificity to improve the power of exploratory studies that eventually lead to clinically relevant findings.

5. **Some of the descriptions are not sufficiently rigorous. For example, "very similar": is there an objective quantification? Is the difference statistically significant?**

   We thank the editor for making this point. For the example in question (i.e., the use of "very similar" in the "Association between gene activity in normal and neoplastic tissue" section), we had provided a quantification via the Spearman rank correlation values in Table 2 and the Euclidean distances in Table 3. We have updated the revised manuscript to reference these

quantitative measures in places where the text makes a qualitative statement such as "very similar". Unfortunately, accurate measures of statistical significance are not feasible for the various correlation estimates. In particular, the rank correlations are computed across all measured genes so the n value is very large and the estimated p-values are all ~ 0. In reality, the effective n value for this correlation computation is smaller given the strong correlations between the genes. However, even if this effective n value could be estimated, the tests would still generate highly significant p-values given the very large number of measured genes. Given these issues, we felt that reporting significance values for these statistics would not be helpful and potentially misleading so have limited the results to the point estimates. We have added some text clarifying the lack of inference results for these correlation estimates in a new Limitations section near the end of the manuscript.

6. **TCGA has more than 21 cancer types. Why select those 21?**

   We thank the editor for raising this question. The 21 TCGA cancer types were selected based on availability of data for the corresponding normal tissues in the Human Protein Atlas. A similar subsetting was performed by Uhlen et al. [1] for 17 cancer types. In contrast to Uhlen et al., we separately analyzed the three renal cancer types (kidney chromophobe, kidney renal clear cell carcinoma, and kidney renal papillary cell carcinoma), separately analyzed colon cancer and rectal cancer, and separately analyzed lung adenocarcinoma and lung squamous cell carcinoma. We have added some additional clarifying text to the revised manuscript to highlight the rationale for the selected cancer types/normal tissues and differences from the Uhlen et al. pairings.

2 Reviewer 1 Comments

Highly interesting work on how best to disentangle cancer-specific processes from tissue-specific ones. With a few revisions to ensure clarity, this paper will be a valuable contribution to the field of cancer transcriptomics.

2.1 Reviewer 1 Major Comments

1. **Major elements of the paper rely on the comparison between cancer and corresponding normal tissues, but it is not discussed in the paper how these pairings were made. Ostensibly the information is based on which tissues were available from HPA, but this needs to be made explicit in the body of the text. Selecting an appropriate normal tissue to compare to is not a trivial problem for many cancer types. For instance, this paper pairs ovarian cancer (OV) with normal ovarian tissue. The overwhelming majority of ovarian cancer cases in TCGA are high-grade serious ovarian cancer, which is believed to frequently arise in the Fallopian tube and more closely resembles normal Fallopian tissue than normal ovarian tissue. The challenge of pairing cancers and normal tissues is highlighted in Table 2, where several cancer types were more correlated with another tissue type than the one selected. It is essential that the pairing decisions are described and justified.**

   We thank the reviewer for raising this important issue. As the reviewer suspected, the pairing was made on the basis of available normal tissues in the HPA and for consistency with the Uhlen et al. work (similar cancer/normal pairing were used in that paper). We have added

additional text to the revised manuscript to clarify the rationale for the pairings and detailed the associated limitations in cases like ovarian cancer and melanoma in a new Limitations section near the end of the manuscript.

2. **While there's an obvious reason to use only the first two principal components when plotting, the distance calculations shown in Table 3 are feasible on a range of principal components and likely to be highly affected by number of PCs used. The paper needs some sort of explanation or justification of the selection of 2 principal components, including percentage of variance explained by PCs 1 and 2 and ideally an elbow plot of percent variance explained by a range of PCs.**

   The reviewer makes an excellent point and, as outlined above in response to a similar comment from the editor, the projection onto the first 2 PCs was used for consistency with the similar analysis by Uhlen et al. [1]. Although use of these PCs for a single visualization makes sense (they explain more gene expression variance than any other pair of PCs), other PCs are also biologically meaningful and Euclidean distances can be computed for all of the PCs. To address this concern, we have included in the supplemental material projections onto PCs 3-8 and a scree plot showing all PC variances. So that the distances in Table 3 mirror the visualized projects, we have kept 2 PC-based distances for this table but have added a version of Table 3 to the supplemental material where the Euclidean distances are computed on all PCs with non-zero variance. As seen from these additional results, cancers and associated normal tissues differ primarily along the directions of the 1st and 3rd PCs, which is consistent with the findings in Table 2 regarding the overall similarity between the transcriptomes of cancers and associated normal tissues. We have updated the relevant text in the main manuscript to clarify the interpretation of the projection onto the first 2 PCs and mention that results for additional PCs can be found in the supplement.

2.2 Reviewer 1 Minor Comments

1. **The manuscript is not organized according to the standards outlined in the PLOS Computational Biology submission guidelines. Reorganizing into the standard sections (Introduction, Results, Discussion, Materials and Methods) could be done without too much alteration of the text itself.**

   We appreciate the suggestion from the reviewer and are happy to make the change if deemed necessary, however, we believe that including a brief Materials and Methods section before the results will make the paper overall much easier to read and understand. In particular, we feel that a short definition of the relevant statistics will help users interpret the presented figures and tables.

2. **The right panel of Figures 1 and 2 is very difficult to interpret, with many overlapping shaded regions. For visual clarity, using lines to connect single cancer-normal tissue pairs and only using shaded ovals for the multi-associations would communicate the results of the figure much more clearly.**

   We thank the reviewer for raising this concern. We have experimented with various approaches for this plot and believe that, while the overlapping shaded regions are not ideal, they do a better job of illustrating the cancer/normal associations than the use of just colored lines, which are very hard to visually distinguish. We have consequently kept these figures unchanged but are open to modifications if this issue is deemed critical.

3. **Normal tissue data from HPA, the file "HPA.normal.FPKM.GDCpipeline.csv" as described in the supplemental methods, is not clearly accessible. A link to this data should be provided.**

   We thank the reviewer for making this suggestion and have created a paper website (https://hrfrost.host.dartmouth.edu/CancerNormal/) where this specially processed HPA data file can be accessed along with files holding the gene-level statistics and all of the logic needed to reproduce the paper results.

3 Reviewer 2 Comments

This article has studied the normal tissue data for the analysis of cancer genomics. Advanced from the existing studies that usually focused on the paired analysis of tumor and adjacent normal samples, this study has explored the genome-wide association between the transcriptomes of 21 TCGA cancer types and their associated normal tissues as profiled in healthy individuals from the Human Protein Atlas. Some interesting findings have been observed, where there is a strong association between tissue-specific and cancer-specific expressions, and this association can be leveraged to improve the prognostic modeling of cancer, the comparative analysis of different cancer types, and the analysis of cancer and normal tissue pairs. Overall, this article is biologically interesting. However, more detailed discussions would be added to improve the presentation. Specifically, I have a number of concerns regarding the paper.

1. **Since the results presented in the paper are based on data from TCGA and HPA, it is suggested to provide more detailed descriptions on these data. For example, for each cancer type or normal tissue, it is better to provide the sample size and dimension of genes. Is any preprocessing conducted on the original downloaded data, such as the matching of the genes in different cancer types or normal tissues, prescreening to reduce the number of genes, or standardization on the gene expression measurements?**

   We thank the reviewer for their suggestion and have included a table in the Supplemental Material that provides the number of measured genes and sample sizes for the analyzed normal tissues and cancer types along with more detail on preprocessing. In brief, genes were filtered to the 19,670 genes with measurements on all normal tissues and cancer types and RNA-seq data was normalized as FPKM+1 using the Genome Data Commons (GDC) pipeline for the TCGA data and using a pipeline similar to GDC pipeline for the HPA data. Sample sizes for the cancers are based on the TCGA data sets. For HPA RNA-seq data, measurements were made on frozen tissue sections from the Uppsala Biobank for three healthy individuals. Although the HPA data was collected on a much smaller number of individuals, the mean gene expression estimated from this data have been shown to provide accurate estimates of gene tissue-specificity as detailed in the original HPA Science paper [2]. We have mentioned the small HPA sample size in the new Limitations section.

2. **PCA is conducted on the matrix consisting of all c_{ij} and n_{ij} statistics, and also that consisting of all $c*_{ij}$ and $n*_{ij}$ statistics, where the sample size is 38. What is the total number of c_{ij} and n_{ij} statistics? The PCA approach may be infeasible, as the number of genes is usually much larger than the sample size (38).**

The reviewer raises good question. In this case, the rank of the sample covariance matrix is 38 (the matrices of n, c, n^*, and c^* statistics have 38 rows and 19,670 columns) so PCA will generate 38 PCs with non-zero variances (i.e., the sample covariance matrix will have 38 non-zero eigenvalues). So, PCA in this scenario is valid although the estimates of PC variances and loadings will have larger variances then would be encountered in a $p < n$ scenario. If the reviewer is curious, Johnstone provides an interesting discussion of the consistency of PCA in different asymptotic scenarios as a motivation for sparse PCA methods [3].

3. **Would you please provide more detailed discussions on the definitions of c^*_{ij} and n^*_{ij} statistics, which are referred to as tissue specific and cancer-specific gene expressions. For example, why log2 transformation is conducted for $c*_{ij}$ and $n*_{ij}$ statistics, but not for c_{ij} and n_{ij} statistics. Why $c*_{ij}$ and $n*_{ij}$ can be referred to as tissue specific and cancer-specific gene expressions? They are in a sense the simple scaled values of c_{ij} and n_{ij} statistics.**

   We thank for the reviewer for raising this issue and have updated the methods section in the main manuscript to provide more detail on the definitions of the $c^*_{i,j}$ and $n^*_{i,j}$. In brief, these capture the relative expression of the gene in a given normal tissue or cancer as compared to the average measured across all of the profiled normal tissues or cancer types. The Human Protein Atlas used a very similar ratio of mean expression in a given tissue to the average mean expression in other tissues to identify tissue-specific genes [2] We applied a log2 transformation to these ratios to provide a roughly symmetric distribution around 0 which has a nice interpretation (this is very similar to the standard practice of generating gene expression log fold-change values). We did not apply a log transformation to the mean expression statistics ($c_{i,j}$ and $n_{i,j}$) so that these kept the original FPKM interpretation (note that the presence or absence of a log2 transformation does not impact the rank correlation estimates). The c^*_{ij} and n^*_{ij} statistics therefore represent the specificity of gene expression in a given tissue/cancer as compared to other tissues/cancers, i.e., large positive values represent genes whose expression in the tissue/cancer is much larger than the average found in other tissues/cancers and large negative values represent genes whose expression in the tissue/cancer is much smaller than the average found in other tissues/cancers. Although the calculation of the statistics is quite simple (as the reviewer noted, they are just scaled values with a gene-dependent scaling factor), they are effective at capturing gene expression tissue/cancer-specificity.

4. **In section "Association between normal tissue-specificity, cancer/normal differential expression and cancer survival", it is demonstrated that "an increase in expression of normal tissue specific genes is associated with improved survival in cancer. Genes that are not tissue-specific have the inverse association, i.e., an increase in expression is associated with worse survival. Genes that are down-regulated in a tissue relative to other tissues tend to have no survival association". Would you provide more detailed discussions on these statements? For example, how can we distinguish genes that are normal tissue specific or not? Why the results in Table 4 can support these statements?**

   We thank the reviewer for their comment and have have updated the revised manuscript to provide a more detailed discussion of these associations between tissue-specificity and cancer survival. On the question of how we can distinguish genes that are tissue specific or not, this can be accomplished via the $n^*_{i,j}$ statistics: large positive values of $n^*_{i,j}$ corresponding to tissue-specific genes, i.e., genes that are more highly expressed in the tissue than in other tissues, large negative values correspond to genes with lower expression in the tissue than

in other tissues and values near 0 correspond to genes that are expressed at a similar level in the tissue as compared to other tissues. We have added text to Table 1 to help clarify this interpretation. The ρ_{surv} correlation coefficient in Table 4 provides general support for this association, i.e., values are either positive or very close to 0 which implies that as tissue-specificity increases genes are more likely to be unfavorably prognostic. A more detailed visualization of this association for each of the analyzed cancer types is provided by Figure 3b for liver cancer and Figure S4 the Supplemental Material for all 21 analyzed cancer types.

5. **"Figure 4. Cell coloring reflects the Pearson correlation between the fold-change in gene expression between each pair of cancers and the corresponding pair of normal tissues" is confusing. It is better to provide more details on the calculation of the corresponding Pearson correlation.**

   We thank the reviewer for raising this concern and have updated the revised manuscript with a more detailed description regarding how the correlation coefficients visualized in Figure 4 are computed.

6. **In section "Using normal tissue gene activity to improve the comparative analysis of cancers", the results support that "when genes exhibiting significant normal tissue DE are removed, the comparative cancer results are distinct from the normal tissue results." However, what is the improvement of using normal tissue gene activity for the comparative analysis of cancers?**

   We thank the reviewer for asking this question. For the scenario illustrated in Figure 4 and Table 5, the challenge being address is that the gene expression differences between cancers typically mirror the gene expression differences found between the corresponding normal tissues, which can make it challenging to identify differences due solely to the distinct mutagenic processes. An example that highlights this is one we explored in an earlier paper involving the comparative analysis of gene expression data from primary colorectal tumors and lung and liver metastatic lesions [4]. Our initial DE gene and pathway analysis between the primary and lung or liver mets found that transcriptomic differences between the primary tumors and mets were dominated by normal tissue-specific genes and pathways, i.e., liver met gene expression differed according to liver-specific genes/pathways and lung met gene expression differed according to lung-specific genes/pathways. When genes/pathways that were specific to normal liver, lung, colon and rectal tissue were removed, however, the primary vs. liver met and primary vs. lung met analyses both captured similar results related to the differences between the primary and metastatic lesions that were independent of the metastatic host tissue. So, the improvement relates to the type of results identified by a comparative transcriptomic analysis rather than an improvement in statistical power (the benefit seen for the cancer vs. normal and survival analysis use cases).

4 Reviewer 3 Comments

1. **More importantly, rudimentary typos (eg. BRAC 1/BRAC2 rather than BRCA1/BRCA2, reverse quotations marks at the start of a quotation in many places) could have been avoided by carefully proofreading before submission.**

   We appreciate the reviewer identifying these typos and apologize for not catching this on the first submission. Unfortunately, a search of the submitted PDF did not reveal a case of "BRAC 1" with a space between "BRAC" and "1" and the reverse quotation marks is

the result of LaTeX formatting. We have done a careful proofreading pass of the revised manuscript to help ensure a minimal number of typos in the manuscript.

2. **In Table 2, what are the sample size, and the distribution of age and gender for each of cancers and HPA tissues? Largely different proportions of those factors in these two groups (cancer and HPA tissue) can lead to very biased or misleading results.**

   We thank the reviewer for raising this issue. Comment # 1 by Reviewer 2 addressed a similar issue. We have included sample size, age and gender information for the TCGA cohorts in Table S1 in the Supplemental Material. For the HPA RNA-seq data, measurements were made on frozen tissue sections from the Uppsala Biobank for three healthy individuals and age and gender information is not available. Although the HPA data was collected on a much smaller number of individuals, the mean gene expression estimated from this data have been shown to provide accurate estimates of gene tissue-specificity as detailed in the original HPA Science paper [2]. While genders will overwhelmingly align for the sex-specific cancers/tissues (cervix, breast, ovary, prostate and testis), we unfortunately cannot comment on gender alignment between the TCGA and HPA samples for other cancers/tissues. We also cannot comment on age alignment. We have mentioned the small HPA sample size and age/gender alignment issues in the new Limitations section. While these issues are certainly limitations of the results presented in this paper, we believe the overall results, which are based on mean expression levels for almost 20k genes, accurately capture the general associations between gene expression in normal tissues and cancers.

3. **The author only reported the Spearman's correlation without the statistical inference (eg. p-value).**

   We thank the reviewer for raising this concern. The editor made a similar comment (see the editor's comment #5). As we outlined in response to the editor's comment, accurate measures of statistical significance are not feasible for the reported correlation estimates. In particular, the rank correlations are computed across all measured genes so the n value is very large and the estimated p-values are all ~ 0. In reality, the effective n value for this correlation computation is smaller given the strong correlations between the genes. However, even if this effective n value could be estimated, the tests would still generate highly significant p-values given the very large number of measured genes. Given these issues, we felt that reporting significance values for these statistics would not be helpful and potentially misleading so have limited the results to the point estimates. We have added some text clarifying the lack of inference results for these correlation estimates in a new Limitations section near the end of the manuscript.

4. **One of the author's key claims in this manuscript is that "majority of the profiled cancers are mostly strongly correlated with their corresponding normal tissue." However, if I understood Table 2 correctly, this seems to be true for only 12 out of 21 cases, and thus the author's claim is not strongly supported.**

   We thank the reviewer for identifying this issue and agree that, while 12 out of 21 does constitute a majority, it is only 57%. Major comment 1 from reviewer 1 identified a related issue regarding the imperfect alignment between cancer types and corresponding normal tissues. We have highlighted this challenge in the new Limitations section. Even when the alignment between cancers and normal tissues is only approximate, we still believe the pattern of gene

expression in the associated normal tissues still provides useful information regarding gene activity in the associated cancer.

5. **For certain gender dominant cancers, eg. prostate and breast, it is quite natural to think that they are more correlated with their corresponding normal tissue, compared to other non-corresponding tissues since all data consists of the same gender, which seemed to be also supported by the results from Table 2. However, gender specificity was ignored in this manuscript. This should not be the issue for the paired tumor/normal analysis, but I think in this aggregated approach, at least important biological factors such as gender should be controlled or matched to infer the role of normal tissues more accurately.**

   The reviewer makes a very good point regarding the impact of gender on the analyses reported in this paper. As detailed in response to comment # 2, we have provided TCGA gender statistics in Table S1. Unfortunately, gender information is not available for the HPA normal tissue gene expression data (though it can be inferred for the gender-specific normal tissues). While we believe that our focus on mean expression values (computed across all samples in an TCGA cohort or across all normal samples from HPA) helps mitigate the potential bias that may be introduced by a gender mismatch between the cancer and normal samples (and a similar strategy was used successfully by Uhlen et al. in both the original Human Protein Atlas paper [2] and Human Pathology Atlas paper [1]), it is an important limitation of this analysis that we have noted in the new Limitations section.

6. **The author advocated the new measures of c* and n*, which is basically simple log transformation of the original cancer and normal tissue specific gene expression, respectively, while subtracting their batch effects. The author argued that after using the new measures, the PCA results indicate that normal tissue and cancer are no longer separated. However, I doubt about the logical reasoning of how the author came to this conclusion. First of all, the usage of different scales of x (and y) axes in the figures 1 and 2 is misleading. Fixing the same scale in both figures, the difference may not be large enough as the author originally considered. Most of all, there is no mathematical or heuristic justification of the new measures of c* and n* regarding why they are better measures than c and n.**

   We thank the reviewer for raising this issue. Regarding the PCA projection and computed distances between cancers and normal tissues, please see our responses to comment #3 from the editor and major comment #2 from reviewer 1. Regarding the biological and mathematical interpretation of the c^* and n^* statistics, please see our response to the similar comment #3 from reviewer 2. While the c^* and n^* statistics are quite simple, i.e., just the log2 fold-change in mean expression in one normal tissue (or cancer) relative to the average mean expression in all tissues/cancers, we believe they effectively capture the specificity of gene expression in that tissue/cancer; a very similar statistic was used successfully by Uhlen et al. in their 2015 [2] and 2017 [1] Science papers to quantify the tissue/cancer-specificity of gene expression.

References

[1] Mathias Uhlen, Cheng Zhang, Sunjae Lee, Evelina Sjöstedt, Linn Fagerberg, Gholamreza Bidkhori, Rui Benfeitas, Muhammad Arif, Zhengtao Liu, Fredrik Edfors, Kemal Sanli, Kalle von

Feilitzen, Per Oksvold, Emma Lundberg, Sophia Hober, Peter Nilsson, Johanna Mattsson, Jochen M Schwenk, Hans Brunnström, Bengt Glimelius, Tobias Sjöblom, Per-Henrik Edqvist, Dijana Djureinovic, Patrick Micke, Cecilia Lindskog, Adil Mardinoglu, and Fredrik Ponten. A pathology atlas of the human cancer transcriptome. *Science*, 357(6352), Aug 2017.

[2] Mathias Uhlén, Linn Fagerberg, Björn M Hallström, Cecilia Lindskog, Per Oksvold, Adil Mardinoglu, Åsa Sivertsson, Caroline Kampf, Evelina Sjöstedt, Anna Asplund, IngMarie Olsson, Karolina Edlund, Emma Lundberg, Sanjay Navani, Cristina Al-Khalili Szigyarto, Jacob Odeberg, Dijana Djureinovic, Jenny Ottosson Takanen, Sophia Hober, Tove Alm, Per-Henrik Edqvist, Holger Berling, Hanna Tegel, Jan Mulder, Johan Rockberg, Peter Nilsson, Jochen M Schwenk, Marica Hamsten, Kalle von Feilitzen, Mattias Forsberg, Lukas Persson, Fredric Johansson, Martin Zwahlen, Gunnar von Heijne, Jens Nielsen, and Fredrik Pontén. Proteomics. tissue-based map of the human proteome. *Science*, 347(6220):1260419, Jan 2015.

[3] Iain M. Johnstone and Arthur Yu Lu. On consistency and sparsity for principal components analysis in high dimensions. *Journal of the American Statistical Association*, 104(486):682–693, June 2009.

[4] Yasmin Kamal, Stephanie L Schmit, Hannah J Hoehn, Christopher I Amos, and H Robert Frost. Transcriptomic differences between primary colorectal adenocarcinomas and distant metastases reveal metastatic colorectal cancer subtypes. *Cancer Res*, 79(16):4227–4241, Aug 2019.
