# Supplementary material for: Analyzing cancer gene expression data through the lens of normal tissue-specificity
Source: PLoS Comput Biol. 2021 Jun 18;17(6):e1009085. doi: 10.1371/journal.pcbi.1009085 (PMC8244857; doi:10.1371/journal.pcbi.1009085)
Supplement: Attachment — Submitted filename: Frost_CancerAndTissueSpecificity_Response_to_Comments.pdf [file pcbi.1009085.s003.pdf]

Response to reviewer comments for "Analyzing cancer gene expression data through the lens of normal tissue-specificity"

H. Robert Frost

We appreciate the helpful feedback from reviewers on our revised manuscript. Responses to each of the reviewer's comments are outlined in the sections below (comments are in bold and our response follows in normal font). In addition to the revised manuscript, the resubmission includes files showing the differences between these revised versions and the original versions, as computed via latexdiff.

1 Editor Comments

The revision is largely satisfactory. One reviewer still has some very minor lingering concerns – please address them properly. Otherwise, congratulations on a job nicely done.

2 Reviewer 1 Comments

The paper is substantially improved compared to the first submission. The previous concerns about justifying number of principal components used have been sufficiently addressed, and the inclusion of a limitations section and guidance to researchers on how to perform similar analyses are beneficial.

2.1 Reviewer 1 Minor Comments

1. **There is still a paucity of citations in the paper. Several statements in the paper ought to be combined with a source, for instance:**

   We thank the reviewer for raising this issue and have added additional citations to the areas noted (see below).

   (a) **Introduction lines 45-48, "While many researchers have investigated the association between normal tissue biology and cancer development in the context of specific cancer types and the associated cancer drivers (e.g., the association between estrogen sensitive tissues, BRAC1/BRAC2 mutations and cancer development)". The relevant work, or at least one or two representative papers from this work, should be cited.**

   We have added citations to Elledge et al. [1], Avraham et al. [2], and Heijink et al. [3] for the association between estrogen sensitive tissues, BRCA1/BRCA2 mutations and cancer development.

(b) **Limitations section lines 289-291, re: how high-grade serous ovarian tumors are thought to originate in the Fallopian tube.**

We have added citations to Labidi-Galy et al. [4] and Mei et al. [5] for studies regarding the Fallopian tube origin of high-grade serous ovarian tumors.

2. **As Reviewer 3 noted, the genes BRCA1/BRCA2 are incorrectly referred to as BRAC1/BRAC2, and this doesn?t appear to have been changed in the resubmission.**

   Thank you for catching this. Despite it being noted by Reviewer 3 and reading over the reference in the paper multiple times, I failed to notice the "CA"/"AC" switch! This has been corrected in the revised manuscript.

3. **The first paragraph of the section "Use of normal tissue-specificity to improve cancer survival analysis", specifically lines 264-268, is slightly jumbled post-revision. Consider revising for clarity.**

   Thank you for identifying this issue. I believe the problem is primarily due to the use of Section references in latex for non-numbered sections. In this case, the entire section titles ("Using normal tissue gene activity to improve the comparative analysis of cancers" and "Use of normal tissue-specificity to improve the comparative analysis of normal and neoplastic tissue") get copied into the manuscript text. The manuscript has been revised to no longer incorporate those full section titles in this paragraph.

3 Reviewer 2 Comments

All my comments have been addressed. I have no further comment.

4 Reviewer 3 Comments

The authors have addressed all the issues raised.

References

[1] Stephen J Elledge and Angelika Amon. The brca1 suppressor hypothesis: an explanation for the tissue-specific tumor development in brca1 patients. *Cancer Cell*, 1(2):129–32, Mar 2002.

[2] Ayelet Avraham, Susanna Feldman, Sean Soonweng Cho, Ayala Kol, Lior Heler, Emmanuela Riklin-Nahmias, Avishay Sella, Tamar Karni, Tanir M Allweis, Saraswati Sukumar, and Ella Evron. Breast-specific epigenetic regulation of deltanp73 and its role in dna-damage-response of brca1-mutated human mammary epithelial cells. *Cancers (Basel)*, 12(9), Aug 2020.

[3] Anne Margriet Heijink, Francien Talens, Lucas T Jae, Stephanie E van Gijn, Rudolf S N Fehrmann, Thijn R Brummelkamp, and Marcel A T M van Vugt. Brca2 deficiency instigates cgas-mediated inflammatory signaling and confers sensitivity to tumor necrosis factor-alpha-mediated cytotoxicity. *Nat Commun*, 10(1):100, 01 2019.

[4] S Intidhar Labidi-Galy, Eniko Papp, Dorothy Hallberg, Noushin Niknafs, Vilmos Adleff, Michael Noe, Rohit Bhattacharya, Marian Novak, Siân Jones, Jillian Phallen, Carolyn A Hruban,

Michelle S Hirsch, Douglas I Lin, Lauren Schwartz, Cecile L Maire, Jean-Christophe Tille, Michaela Bowden, Ayse Ayhan, Laura D Wood, Robert B Scharpf, Robert Kurman, Tian-Li Wang, Ie-Ming Shih, Rachel Karchin, Ronny Drapkin, and Victor E Velculescu. High grade serous ovarian carcinomas originate in the fallopian tube. *Nat Commun*, 8(1):1093, 10 2017.

[5] Jie Mei, Huixiang Tian, Hsuan-Shun Huang, Che-Fang Hsu, Yuligh Liou, Nayiyuan Wu, Wei Zhang, and Tang-Yuan Chu. Cellular models of development of ovarian high-grade serous carcinoma: A review of cell of origin and mechanisms of carcinogenesis. *Cell Prolif*, 54(5):e13029, May 2021.
